# Analysis of *ATF6* and *PLAT* Expressions in Relation to hsa-miR-340-5p in Childhood Obesity

**DOI:** 10.3390/ijms27062606

**Published:** 2026-03-12

**Authors:** Yaşar Topal, Tuba Edgünlü, Dilek Akbaş, Çilem Özdemir, Hatice Topal, Habip Almiş, Ecenur Özdemir

**Affiliations:** 1 Department of Child Health and Diseases, Sincan Training and Research Hospital, 06000 Ankara, Turkey; haticetopaldr@gmail.com (H.T.); drhabipalmis@gmail.com (H.A.); 2 Department of Medical Biology, Faculty of Medicine, Muğla Sıtkı Koçman University, 48000 Muğla, Turkey; tedgunlu@gmail.com (T.E.); ozdemirnurece@gmail.com (E.Ö.); 3 Department of Molecular Biology and Genetics, Graduate School of Natural and Applied Sciences, Muğla Sıtkı Koçman University, 48000 Muğla, Turkey; dilekakbas027@gmail.com; 4 Department of Bioinformatics, Graduate School of Natural and Applied Sciences, Muğla Sıtkı Koçman University, 48000 Muğla, Turkey; cilemmozdemir@gmail.com

**Keywords:** childhood obesity, endoplasmic reticulum stress, *ATF6*, fibrinolysis, tissue-type plasminogen activator, cardiovascular risk, microRNA, metabolic dysfunction

## Abstract

Childhood obesity is a complex pathology that triggers early vascular damage through endoplasmic reticulum (ER) stress and fibrinolytic imbalance; however, the role of the *ATF6/PLAT* regulatory axis in this process has not yet been fully elucidated. This study aims to investigate the molecular basis of vascular risk by determining the expression levels of these genes and the potential regulatory hsa-miR-340-5p in children with obesity. Gene expression analyses were performed using the RT-qPCR method on blood samples obtained from 55 children with obesity and 40 healthy controls, while in silico protein–protein interaction (PPI) networks were mapped using the STRING database. The findings revealed that *ATF6* expression was significantly downregulated (*p* < 0.001) and *PLAT* expression was significantly upregulated (*p* = 0.005) in the obese group compared to controls. No significant difference was detected in hsa-miR-340-5p levels (*p* = 0.447). PPI analysis confirmed the strong functional clustering of *ATF6* with metabolic stress pathways and *PLAT* with coagulation cascades. In conclusion, the suppression of *ATF6* in obesity indicates the “exhaustion” of adaptive cellular defense mechanisms, while the upregulation of *PLAT* points to a compensatory response to the chronic prothrombotic environment. These molecular alterations demonstrate that vascular risk in childhood obesity begins at the transcriptomic level long before clinical symptoms emerge, highlighting the *ATF6/PLAT* axis as a potential biomarker for early risk assessment.

## 1. Introduction

**Figure 1:**
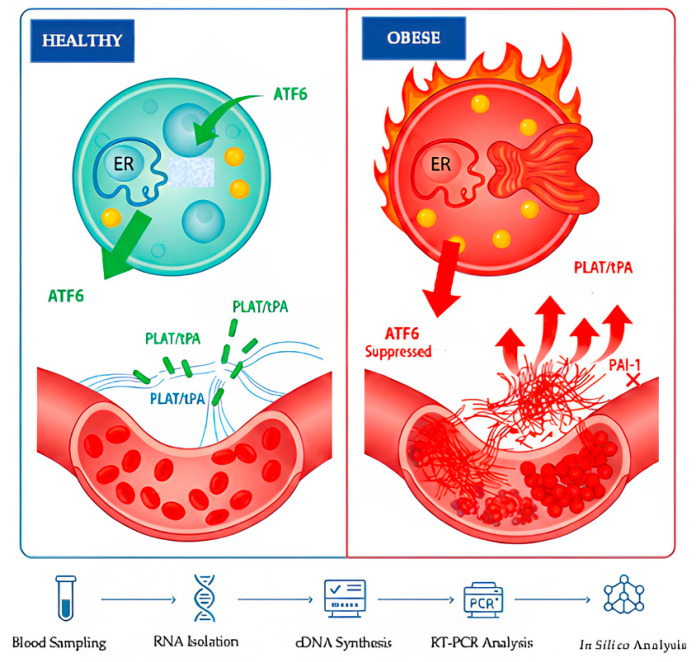
Experimental workflow and graphical summary of molecular mechanisms in childhood obesity. The study encompasses the following processes: venous blood sampling, RNA isolation, cDNA synthesis, RT-qPCR for gene expression analysis, and in silico protein-protein interaction (PPI) mapping via the STRING database. In healthy controls, endoplasmic reticulum (ER) homeostasis and fibrinolytic balance are maintained. In the obese group, chronic metabolic stress leads to decreased *ATF6* expression (exhaustion of adaptive UPR mechanisms). In contrast, the significant increase in *PLAT* (tPA) expression represents a compensatory fibrinolytic response to the prothrombotic environment in pediatric obesity.

Childhood obesity is increasingly recognized as a complex multifactorial pathology rather than a simple consequence of excess weight [1,2]. In addition to genetic predisposition, contemporary sedentary lifestyles and dysregulated dietary habits have transformed childhood obesity into a critical public health concern [1,2]. These environmental and behavioral factors culminate in a chronic state of positive energy balance, which not only drives adipose tissue expansion but also perturbs cellular homeostasis, thereby precipitating a spectrum of metabolic comorbidities [3]. Central to this cellular dysfunction is the impairment of the endoplasmic reticulum (ER), often referred to as the cell’s ‘quality control center’ [4]. Under conditions of chronic nutrient oversupply and consequent insulin resistance, the protein-folding capacity of the ER is overwhelmed, precipitating a state of cellular dysfunction known as Endoplasmic Reticulum (ER) stress [4,5]. In response to this proteotoxic stress, the cell activates an adaptive signaling pathway known as the Unfolded Protein Response (UPR) [3,6]. Among the canonical UPR transducers, Activating Transcription Factor 6 (*ATF6*) initially fulfills a protective function by upregulating chaperone genes to restore ER proteostasis (Figure 1) [7]. Conversely, under the persistent strain of chronic metabolic overload characteristic of obesity, this adaptive mechanism falters, and the sustained activation of *ATF6* undergoes a deleterious functional shift [7]. While primarily acting as a transcriptional activator, its prolonged signaling has been implicated in the exacerbation of hepatic steatosis and the worsening of systemic insulin resistance [5,7].

Crucially, this disruption of lipid metabolism and ER function is not confined to tissue-specific stress but acts as a precursor to systemic vascular inflammation and endothelial dysfunction [8]. In this context, *ATF6* functions not only as a stress sensor but also as a direct transcriptional regulator of the Plasminogen Activator, Tissue Type (*PLAT*) gene, which encodes the primary enzyme responsible for intravascular fibrinolysis [7]. Although these molecular mechanisms are broadly characterized in adult models, clinical evidence indicates that hemostatic disturbances, particularly impaired fibrinolysis and endothelial activation, manifest early in the clinical course of pediatric obesity [9]. Under physiological conditions, *PLAT*-derived tissue plasminogen activator (tPA) maintains vascular patency by converting plasminogen to plasmin; however, in the obese state, this protective pathway is frequently inhibited by elevated levels of Plasminogen Activator Inhibitor-1 (*PAI-1*), which is secreted abundantly by both dysfunctional adipose tissue and hepatocytes [10,11,12]. Mechanistically, the binding of *PAI-1* to the Low-Density Lipoprotein Receptor-Related Protein 1 (*LRP1*) interferes with the downstream PKA/CREB1 signaling axis, specifically by reducing the phosphorylation of *CREB1*, which in turn diminishes the transcriptional activation of *PLAT* [7,12].

The regulation of this critical balance between ER stress and fibrinolysis extends beyond transcriptional control to include post-transcriptional modulation by microRNAs (miRNAs) [13,14]. Because these small non-coding RNAs can simultaneously target multiple components of the UPR and hemostatic systems, they represent key functional links between metabolic dysfunction and thrombotic risk [15]. Within this regulatory network, hsa-miR-340-5p has emerged as a significant candidate for investigation, particularly due to bioinformatic evidence suggesting its potential involvement in the modulation of UPR-related pathways and fibrinolytic gene expression. Given the complex interplay between cellular stress and hemostatic imbalance, this study aimed to evaluate the expression profiles of *ATF6*, *PLAT*, and hsa-miR-340-5p in children with obesity compared to a healthy control group.

## 2. Results

### 2.1. Demographic and Clinical Characteristics of Obese and Control Groups

A total of 95 participants were included in the study, comprising 55 obese and 40 control individuals. The demographic and clinical characteristics of the study population are summarized in Table 1.

There was no statistically significant difference in age between the obese and control groups (*p* = 0.341). Similarly, birth weight did not differ significantly between groups (*p* = 0.755). As expected, body mass index (BMI) was significantly higher in the obese group compared with controls (*p* < 0.001). The obese group also exhibited a significantly longer sleep duration (*p* = 0.042) and greater daily mobile screen time (*p* = 0.010). No significant difference was observed between the groups in terms of television viewing time (*p* = 0.140). Regarding gender distribution, the proportion of males and females was comparable between the obese and control groups (*p* = 0.548).

### 2.2. Expression Analysis of Target Genes and miRNA

**Figure 2:**
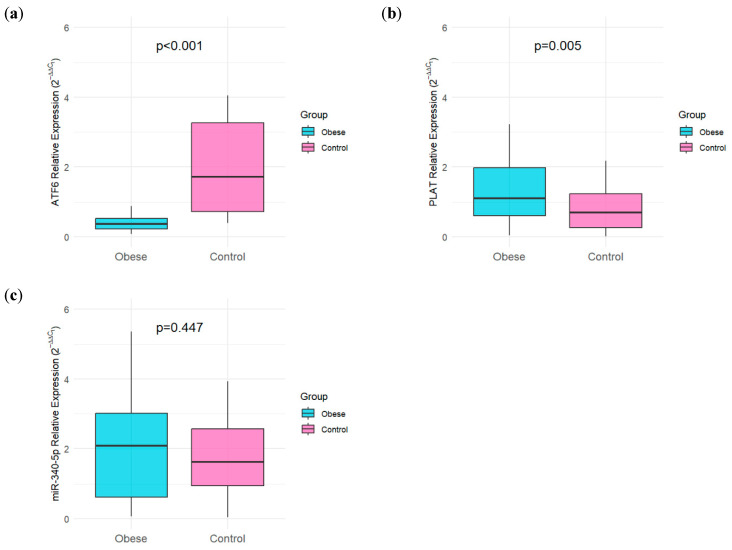
Comparative analysis of gene and miRNA expression levels. (**a**) *ATF6* expression significantly decreased in the obese group (*p* < 0.001). (**b**) *PLAT* expression was significantly higher in obese patients compared to controls (*p* = 0.005). (**c**) No significant difference was observed in miR-340-5p expression levels (*p* = 0.447). Box plots represent the median and interquartile range (IQR), with whiskers showing the range. Relative expression levels were normalized using the 2^−ΔΔCT^ method [16].

The relative expression levels of the target genes and miRNA were normalized using the 2^−ΔΔCT^ method, and comparisons between groups were performed using the Mann–Whitney U test. A significant downregulation of *ATF6* was observed in the obese group compared to the control group (*p* < 0.001). Conversely, *PLAT* expression levels were significantly higher in obese patients than in healthy controls (*p* = 0.005). However, no statistically significant difference was found between the groups regarding miR-340-5p expression levels (*p* = 0.447). These results indicate that while *ATF6* and *PLAT* expressions are robustly associated with obesity status, miR-340-5p expression does not exhibit a similar clinical correlation in this specific study population (Table 2 and Figure 2).

### 2.3. Bioinformatic Assessment of Protein Interactions

To further investigate the functional landscape of *PLAT* and *ATF6*, we performed protein–protein interaction (PPI) and enrichment analyses using the STRING database. Details and enrichment results for Network 1 and Network 2 are presented in Table 3. The *PLAT*-centered network (Network 1) revealed a strong association with hemostatic processes. Specifically, *PLAT* was found to cluster with ANXA2, PLG, SERPINE1, SERPINF2, and SERPINB2 within the fibrinolysis. The regulation of plasminogen activation was further supported by the interactions between S100A10, ANXA2, PLG, SERPINE1, and SERPINF2. Additionally, the network showed significant involvement in Complement and coagulation cascades, represented by the core group of *PLAT*, PLG, SERPINE1, SERPINF2, and SERPINB2.

The *ATF6*-centered network (Network 2) demonstrated a robust profile related to cellular stress and metabolic regulation. A comprehensive set of proteins, including VAPB, XBP1, DDIT3, MBTPS2, HSPA5, MBTPS1, ERN1, and EIF2AK3, was identified as part of the Endoplasmic Reticulum (ER) unfolded protein response [17]. Within this network, the ER-nucleus signaling pathway was specifically mediated by the interaction of *ATF6*, XBP1, DDIT3, MBTPS2, HSPA5, MBTPS1, and EIF2AK3. Also, the analysis linked ERN1, XBP1, DDIT3, and EIF2AK3 to Non-alcoholic fatty liver disease (NAFLD), suggesting a metabolic dimension to the *ATF6* interactome. These findings collectively indicate that *PLAT* and *ATF6* serve as critical nodes in fibrinolytic activity and ER-stress management, respectively, through their interactions with these specialized protein clusters.

## 3. Discussion

This study provides the first clinical evidence regarding the interplay between ER stress markers and the fibrinolytic system in pediatric obesity. Our primary findings reveal a significant downregulation of *ATF6* and a concurrent upregulation of *PLAT* in children with obesity compared to healthy controls. These results clinically validate the hypothesis that chronic metabolic stress disrupts the adaptive UPR capacity and alters fibrinolytic balance even at an early age, consistent with recent studies linking ER stress to metabolic dysregulation [8,11]. However, contrary to our in silico predictions, circulating miR-340-5p levels did not show a significant difference between groups, nor did they correlate with target gene expression. This discrepancy suggests that while the bioinformatic analysis correctly identified a potential regulatory network, the systemic reflection of this axis in pediatric circulation may be masked by tissue-specific mechanisms or the dominance of other regulatory miRNAs in the early stages of obesity [12].

### 3.1. The Paradox of ATF6 Decline in Chronic Obesity

One of the most striking findings of our study is the marked suppression of *ATF6* expression in the obese group. Classically, *ATF6* is recognized as a primary sensor of the UPR, typically upregulated to enhance chaperone capacity during acute ER stress [7]. However, our data align with the concept of “UPR exhaustion” or maladaptive remodeling under conditions of chronic metabolic stress. It is well-established that while acute lipotoxicity triggers a protective UPR surge, prolonged exposure to excess nutrients, as seen in childhood obesity, can lead to the attenuation of canonical UPR branches by exceeding the protein folding capacity [18]. The significant decrease in *ATF6* observed in our cohort likely reflects a failure of cellular quality control mechanisms to sustain an adaptive response against persistent stress. This downregulation is critical because *ATF6* deficiency has been linked to the deepening of insulin resistance and systemic inflammation, rather than resolving the proteotoxic load [8,19]. Furthermore, recent pediatric evidence suggests that such ER stress alterations are not merely cellular responses but key contributors to the pathogenesis of metabolic complications in children [20].

### 3.2. Fibrinolytic Imbalance and PLAT Increase

In contrast to the suppression of the UPR sensor, *PLAT* (tPA) expression levels were significantly elevated in the obese group. Under physiological homeostasis, *ATF6* acts as a positive transcriptional regulator of *PLAT*, maintaining basal fibrinolytic capacity [12]. However, the inverse relationship observed in our study, downregulated *ATF6* versus upregulated *PLAT*, suggests a “decoupled” regulatory mechanism specific to the obese state. We hypothesize that this elevation in *PLAT* represents a compensatory response to the chronic pro-thrombotic environment characterized by elevated PAI-1 levels. Recent studies in 2025 confirm that in obesity-induced endothelial dysfunction, PAI-1 is not merely an inhibitor but acts as a signaling molecule that can paradoxically induce *PLAT* expression via the PAI-1/LRP1/PKA/CREB1 axis to prevent thrombotic occlusion [11,21]. Furthermore, chronic low-grade inflammation (e.g., TNF-α and IL-6 signaling) has been shown to independently drive endothelial tPA release, bypassing the exhausted ER stress pathway [21,22]. This “fibrinolytic struggle” demonstrates that obese children are already exhibiting subclinical molecular signs of vascular stress and thrombosis risk.

### 3.3. The Effect of miRNAs and Lifestyle Factors

Although our in silico models identified hsa-miR-340-5p as a potential regulator, our experimental data did not show a significant correlation between this miRNA and its target genes. This discrepancy highlights the complexity of epigenetic regulation in childhood obesity, suggesting that tissue-specific interactions or other dominant metabolic miRNAs may override this axis in systemic circulation. More importantly, our demographic analysis revealed a significant increase in mobile screen time in the obese group. We propose that this sedentary behavior acts as a critical environmental trigger for chronic low-grade inflammation [21]. Sustained inflammatory signaling is known to deepen cellular ER stress, thereby contributing to the “exhaustion” of *ATF6* and the disruption of fibrinolytic balance observed in our study [22].

### 3.4. Strengths and Limitations

This study provides crucial clinical evidence regarding the molecular fingerprints of early-stage childhood obesity, distinguishing itself from preclinical animal models. Validating our experimental findings with STRING database analysis has further clarified the functional clustering between ER stress failure and coagulation cascades. However, the cross-sectional design limits our ability to establish a definitive causal relationship between *ATF6* downregulation and *PLAT* upregulation. Additionally, while transcriptional changes provide immediate insight into cellular reprogramming, future studies incorporating circulating protein levels (tPA and PAI-1) would offer a more comprehensive picture of the translational impact on fibrinolytic potential.

## 4. Materials and Methods

**Figure 3:**
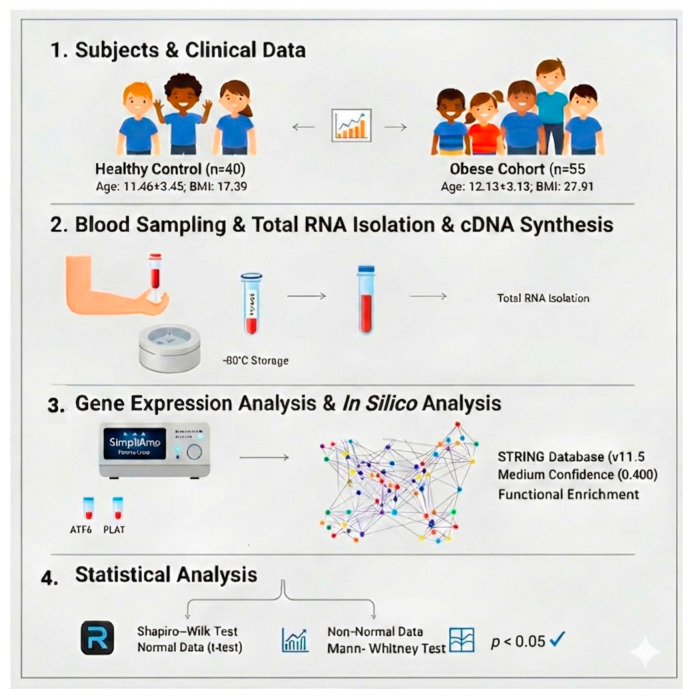
The workflow involves collecting blood samples from obese (*n* = 55) and healthy (*n* = 40) children, followed by RNA isolation and analysis of *ATF6*, *PLAT*, and regulatory miRNAs via RT-qPCR. Functional validation was performed using in silico STRING PPI (protein–protein interaction) networks. The model demonstrates that obesity-induced chronic metabolic stress leads to decreased *ATF6* expression (exhaustion of the adaptive UPR mechanism) and a compensatory increase in *PLAT* expression as a response to the prothrombotic environment in pediatric patients.

The overall experimental workflow, including subject recruitment, molecular procedures, and bioinformatics analysis, is summarized in Figure 3.

### 4.1. Ethics Committee Approval

Ethical approval for this study was obtained from the Scientific Research Ethics Committee of Sincan Training and Research Hospital (Approval Code: BAEK-2025-41).

### 4.2. Subjects

Following approval of the protocol by the Ethics Committee of Sincan Training and Research Hospital, the study was conducted in accordance with the Declaration of Helsinki. Informed consent was obtained from the parents of all participants. The study group consisted of 55 children and adolescents with obesity (OW/OB; Age: 12.13 ± 3.13 years; BMI: 27.91 [25.30–31.48] kg/m^2^) and 40 age- and gender-matched healthy controls (Age: 11.46 ± 3.45 years; BMI: 17.39 [16.20–21.16] kg/m^2^) (Figure 3). CDC growth curves were used to group participants. Exclusion criteria for both groups included a history of chronic metabolic/endocrine diseases (e.g., diabetes, hypothyroidism, Cushing’s syndrome), genetic obesity syndromes (e.g., Prader–Willi syndrome), active infection, and the use of medications affecting body weight within the last three months. Anthropometric data were collected following standard procedures.

### 4.3. Blood Sampling

Venous blood samples (2 mL) were collected in vacuum tubes containing K2-EDTA. The samples were immediately centrifuged at 3000 rpm for 10 min at 4 °C. The resulting plasma and cell pellets were stored at −80 °C until the analysis stage.

### 4.4. Gene Expression Analysis

Total RNA isolation, including the small RNA fraction, was performed using the TRIzol™ Reagent RNA isolation kit (Thermo Fisher Scientific, Carlsbad, CA, USA; Cat No: 15596026) according to the manufacturer’s instructions. Complementary DNA (cDNA) synthesis was performed from 1 µg of total RNA using the cDNA Reverse Transcription Kit (NucleoGene (Istanbul, Turkey; Cat. No: NGMM007)) according to the manufacturer’s protocol [23]. The expression levels of the *ATF6* and *PLAT* genes and hsa-miR-340-5p were determined using the quantitative reverse transcription-polymerase chain reaction (RT-qPCR) method. The RT-qPCR analysis was performed using an automated thermal cycler (SimpliAmp Thermal Cycler, Thermo Fisher Scientific, Carlsbad, CA, USA; Cat No: 15596026) and SYBR Green reagent (NucleoGene, Istanbul, Turkey; Cat. No: NGMM007) in a final volume of 20 µL. *GAPDH* and *U6* were used as internal controls for the normalization of mRNA and miRNA expression levels, respectively [24]. Relative expression levels were calculated using the 2^−ΔΔCt^ method. To ensure technical reproducibility, all measurements were performed in triplicate.

### 4.5. In Silico Analysis

To complement our experimental observations, we conducted in silico analyses. Potential miRNA targets regulating the *ATF6* and *PLAT* genes were predicted using the miRDB online database (http://mirdb.org; accessed on 5 January 2026), utilizing an algorithm for miRNA target prediction and functional annotation [25]. Additionally, protein–protein interaction (PPI) networks for *PLAT* and *ATF6* proteins were mapped using the STRING database (https://string-db.org/; accessed on 5 January 2026) [26]. The analysis was performed using default parameters with a medium confidence score (0.400) to identify functional enrichment of biological pathways.

### 4.6. Statistical Analysis

All statistical analyses were performed using R software (version 4.1.3). Continuous variables were first assessed for normality using the Shapiro–Wilk test [27]. Variables showing a normal distribution were presented as Mean ± Standard Deviation (SD) and compared using the Independent Samples *t*-test [28]. Non-normally distributed data were presented as Median and Interquartile Range (IQR), and group comparisons were conducted using the Mann–Whitney U test. Categorical variables were expressed as number and percentage (n, %) and compared using the Chi-square test. A *p*-value < 0.05 was considered statistically significant.

## 5. Conclusions

In summary, our study demonstrates that childhood obesity is characterized by the exhaustion of the *ATF6*-mediated ER stress response and a concurrent upregulation of the *PLAT* gene. This distinct molecular signature indicates that the cellular “quality control” system is compromised, triggering a compensatory fibrinolytic response aimed at protecting vascular health. Our findings emphasize that obesity-induced vascular stress begins at the transcriptomic level during childhood, preceding the onset of clinical symptoms. Consequently, the potential use of *ATF6* and *PLAT* expression levels as biomarkers for early cardiovascular risk assessment in the pediatric population should be supported by future longitudinal studies.

## Figures and Tables

**Table 1:** Comparison of demographic and clinical characteristics between obese and control groups.

Variable	Obese (*n* = 55)	Control (*n* = 40)	*p* * Value
**Age (years)**	12.13 ± 3.13	11.46 ± 3.45	0.341 *
**Birth weight (kg)**	3.20 (3.00–3.60)	3.20 (3.00–3.50)	0.755 **
**Body Mass Index (BMI)**	27.91 (25.30–31.48)	17.39 (16.20–21.16)	<0.001 **
**Sleep duration (hours/day)**	9.00 (8.00–10.00)	9.00 (8.00–9.00)	0.042 **
**Television viewing time (hours/day)**	2.00 (1.00–3.00)	2.00 (1.00–2.00)	0.140 **
**Mobile screen time (hours/day)**	5.00 (3.00–6.00)	3.00 (2.00–5.00)	0.010 **
**Gender n (%)**			
**Male**	28 (50.9)	17 (42.5)	0.548 ***
**Female**	27 (49.1)	23 (57.5)	

BMI: Body Mass Index; IQR: Interquartile range. Data are presented as Mean ± SD or Median (IQR) where appropriate. * Independent sample *t*-test, ** Mann–Whitney U test, *** Chi-square test. Bold values indicate statistical significance (*p* < 0.05).

**Table 2:** Comparison of Gene and miRNA Expression Levels.

Gene/miRNA	Obese (*n* = 55)	Control (*n* = 40)	*p* * Value
*ATF6*	0.43 (0.23–1.20)	1.28 (0.68–2.40)	**<0.001**
*PLAT*	0.97 (0.59–1.83)	0.69 (0.26–1.23)	**0.005**
miR-340-5p	2.08 (0.62–3.01)	1.63 (0.94–2.57)	0.447

* Mann–Whitney U test, IQR: Interquartile range. Values represent median (IQR) of relative expression levels calculated using the 2^−ΔΔCT^ method. Bold values indicate statistical significance (*p* < 0.05).

**Table 3:** Functional enrichments of networks based on *ATF6* and *PLAT* interactions.

	Network	Proteins	Network Stats	Database	Description	Strength ^1^	FDR ^2^
**1**	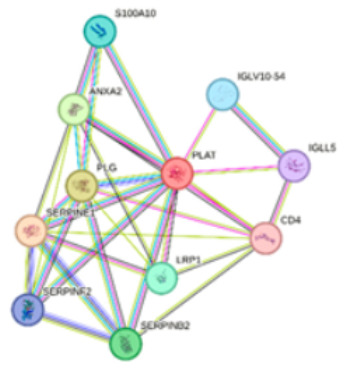	PLAT S100A10 ANXA2 IGLV10-54 IGLL5 PLG CD4 LRP1 SERPINE1 SERPINF2 SERPINB2	number of nodes: 11 number of edges: 31 average node degree: 5.64 avg. local clustering coefficient: 0.8 expected number of edges: 12 PPI enrichment *p*-value: 5.17 × 10^−6^	Gene Ontology	1# GO:0042730 Fibrinolysis 2# GO:0010755 Regulation of plasminogen activation	2.75 2.7	1.57 × 10^−11^ 1.96 × 10^−9^
				KEGG Pathways	1# hsa04610 Complement and coagulation cascades	2.04	2.27 × 10^−7^
**2**	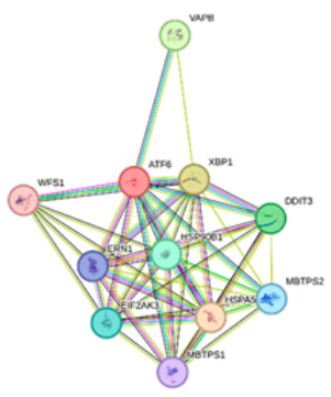	ATF6 VAPB XBP1 DDIT3 MBTPS2 HSPA5 MBTPS1 HSP90B1 ERN1 EIF2AK3	number of nodes: 11 number of edges: 44 average node degree: 8 avg. local clustering coefficient: 0.925 expected number of edges: 12 PPI enrichment *p*-value: 1.26 × 10^−12^	Gene Ontology	1# GO:0030968 Endoplasmic reticulum unfolded protein response 2# GO:0006984 ER-nucleus signaling pathway	2.52 2.64	1.07 × 10^−20^ 6.23 × 10^−17^
				KEGG Pathways	1# hsa04932 Non-alcoholic fatty liver disease	1.69	6.91 × 10^−5^

**Notes:** Nodes represent proteins and edges represent protein–protein associations. Colored nodes indicate query proteins and first shell interactors, while white nodes indicate second shell interactors. Edge colors represent different types of interaction evidence: cyan (curated databases), pink (experimental), green (gene neighborhood), red (gene fusions), blue (gene co-occurrence), light green (textmining), black (co-expression), and light blue (protein homology). **^1^ Strength:** Log10(observed/expected). This measure describes how large the enrichment effect is. It’s the ratio between (i) the number of proteins in your network that are annotated with a term and (ii) the number of proteins that we expect to be annotated with this term in a random network of the same size. **^2^ False Discovery Rate:** This measure describes how significant the enrichment is. Shown are *p*-values corrected for multiple testing within each category using the Benjamini–Hochberg procedure.

## Data Availability

The data presented in this study are available on request from the corresponding author. The data are not publicly available due to privacy and ethical restrictions regarding the pediatric population.

## Abbreviations

The following abbreviations are used in this manuscript:

**ATF6**	Activating Transcription Factor 6
**BMI**	Body Mass Index
**cDNA**	Complementary DNA
**CDC**	Centers for Disease Control and Prevention
**CREB1**	cAMP Response Element-Binding Protein 1
**ER**	Endoplasmic Reticulum
**GAPDH**	Glyceraldehyde-3-Phosphate Dehydrogenase
**IQR**	Interquartile Range
**LRP1**	Low-Density Lipoprotein Receptor-Related Protein 1
**miRNA**	MicroRNA
**NAFLD**	Non-Alcoholic Fatty Liver Disease
**OB**	Obesity
**PAI-1**	Plasminogen Activator Inhibitor-1
**PKA**	Protein Kinase A
**PLAT**	Plasminogen Activator, Tissue Type
**PPI**	Protein–Protein Interaction
**RT-qPCR**	Quantitative Reverse Transcription-Polymerase Chain Reaction
**SD**	Standard Deviation
**tPA**	Tissue-type Plasminogen Activator
**UPR**	Unfolded Protein Response
**WHO**	World Health Organization
